# Women have a higher risk of hospital admission associated with hyponatremia than men while using diuretics

**DOI:** 10.3389/fphar.2024.1409271

**Published:** 2024-08-06

**Authors:** L. C. Hendriksen, M. S. Mouissie, R. M. C. Herings, P. D. van der Linden, L. E. Visser

**Affiliations:** ^1^ Department of Epidemiology, Erasmus MC, University Medical Center Rotterdam, Rotterdam, Netherlands; ^2^ Department of Pharmacy, Tergooi MC, Hilversum, Netherlands; ^3^ School of Pharmacy, Utrecht University, Utrecht, Netherlands; ^4^ PHARMO Institute for Drug Outcomes Research, Utrecht, Netherlands; ^5^ Department of Epidemiology and Data Science, Amsterdam UMC, Location VUmc, Amsterdam, Netherlands; ^6^ Department of Clinical Pharmacy, Haga Teaching Hospital, The Hague, Netherlands; ^7^ Department of Hospital Pharmacy, Erasmus MC, University Medical Center Rotterdam, Rotterdam, Netherlands

**Keywords:** pharmacoepidemiology, sex differences, adverse drug reactions, diuretics, hyponatremia

## Abstract

**Background:**

Hyponatremia is a common electrolyte disturbance and known adverse drug reaction of diuretics. Women tend to be more susceptible for diuretic associated hyponatremia. The aim of this study was to find more evidence whether women have a higher risk of diuretic associated hyponatremia than men measured at hospital admission for specific diuretic groups and whether there is a sex difference in risk of severity of hyponatremia.

**Methods:**

All patients using a diuretic and admitted for any reason to Tergooi MC and Haga Teaching hospital in the Netherlands between the 1st of January 2017 and the 31st of December 2021, with recorded sodium levels at admission were included in this study. Cases were defined as patients with a sodium level <135 mmol/L, while control patients had a sodium level ≥135 mmol/L at admission. Logistic regression analysis was used to calculate odds ratios (OR) with 95% CIs for women versus men and adjusted for potential confounding covariables (age, body mass index, potassium serum level, systolic and diastolic blood pressure, estimated glomerular filtration rate, number of diuretics, comedications and comorbidities). Stratified analyses were conducted for specific diuretic groups (thiazides, loop diuretics and aldosterone antagonists), and adjusted for dose. Furthermore, stratified analyses were performed by severity of hyponatremia (severe: <125 mmol/L), mild: 125–134 mmol/L).

**Results:**

A total of 2,506 patients (50.0% women) were included, of which 516 had hyponatremia at admission (20.6%, 56.2% women). Women had a statistically significantly higher risk for hyponatremia at admission than men (OR 1.37; 95% CI 1.12–1.66) and after adjustment for potential risk factors (ORadj 1.55; 95% CI 1.22–1.98). Stratified analyses showed increased odds ratios for thiazides (ORadj 1.35; 95% CI 1.00–1.83) and loop diuretics (ORadj 1.62; 95% CI 1.19–2.19) among women. Use of aldosterone antagonists was also increased but not statistically significant (ORadj 1.15; 95% CI 0.73–1.81). Women had a statistically higher risk to develop mild and severe hyponatremia than men (ORadj 1.36; 95% CI 1.10–1.68 and ORadj 1.96; 95%CI 1.04–3.68, respectively).

**Conclusion:**

Women have a higher risk of a hospital admission associated with hyponatremia while using diuretics than men. Further research is necessary to provide sex-specific recommendations.

## Introduction

Hyponatremia is a common electrolyte disturbance that is known for many years as an adverse reaction of diuretics (Hwang and Kim, 2010; Egom et al., 2011; Roush and Sica, 2016; Fedrizzi et al., 2018; Nadal et al., 2018; Yamazoe et al., 2018; Filippone et al., 2020). Hyponatremia is defined as having a serum sodium level below 135 mmol/L which is associated with the mechanism of action of diuretics. Diuretics can be divided by their pharmacological mechanism of action into the following groups: thiazides, loop diuretics and aldosterone antagonists. Thiazides and thiazide-like diuretics inhibit the sodium chloride (NaCl)-cotransporter in the distal convoluted tubule (DCT), which causes inhibition of NaCl reabsorption. The diuretic effect is limited, because approximately 90% of the filtered sodium is already reabsorbed before reaching the DCT (UpToDate, 2023; KNMP, 2024). Loop diuretics inhibit the Na-K-Cl-cotransporter in the thick ascending loop of Henle, which causes inhibition of NaCl reabsorption. They promote the excretion of potassium, calcium and magnesium. The diuretic effect of loop diuretics is fast, short and strong (UpToDate, 2023; KNMP, 2024). Aldosterone antagonists inhibit the exchange of sodium and potassium in the collecting duct by blocking the sodium channels or antagonism of aldosterone. Aldosterone antagonists have a weak diuretic effect, since only a small percentage of the filtered sodium reaches the collecting duct (UpToDate, 2023; KNMP, 2024).

Symptoms of hyponatremia encompass falls, deterioration, confusion, or in more severe cases unconsciousness and seizures (Barber et al., 2015; Ramírez et al., 2019). Previous studies described multiple risk factors, for example, sex, age, renal function and hypokalemia for developing diuretic-induced hyponatremia (Chow et al., 2003; Association NIVDI, 2012; Rodenburg et al., 2013; Yamazoe et al., 2018).

Women tend to be more at risk for the adverse drug reactions mentioned above than men (Rodenburg et al., 2011; Hendriksen et al., 2021). Previous studies suggest that these adverse effects may be explained by a higher susceptibility of women to develop diuretic associated hyponatremia (Rodenburg et al., 2011; Rodenburg et al., 2013; Yamazoe et al., 2018; Hendriksen et al., 2021; Hendriksen et al., 2023). It remains, however, unclear why women are more at risk. In literature, these differences are explained by differences in pharmacokinetic factors, like volume of distribution and clearance (Rodenburg et al., 2011; Hendriksen et al., 2023). Women have a lower glomerular filtration rate, lower muscle mass, lower protein intake and lower renal prostaglandin synthesis (Frenkel et al., 2015). This may result in a decreased water excretion compared to men and therefore causes a relative water excess in women.

As noted, previous studies identified that women have an increased risk of hyponatremia associated hospital admissions while using diuretics compared to men (Rodenburg et al., 2011; Rodenburg et al., 2013; Yamazoe et al., 2018; Hendriksen et al., 2021; Hendriksen et al., 2023). The studies showed a range in risk, methods and suggested explanations for this risk. For example, most of these studies only included thiazide users to study the increased risk of hyponatremia. Loop diuretics and aldosterone antagonists were not always included, because they are less likely to cause hyponatremia than thiazides (KNMP, 2013; Sterns, 2020). Furthermore, previous studies used different approaches of diagnosing hyponatremia. In several studies hyponatremia had been recognized as an adverse drug reaction by the treating doctor and coded afterwards (Rodenburg et al., 2011; Hendriksen et al., 2021; Hendriksen et al., 2023). In addition, the studies used different covariables to control for bias in order to estimate the adjusted risk of hyponatremia. For example, not every study adjusted for the same comorbidities, co-medication and patient characteristics [e.g., age, body-mass index (BMI), estimated glomerular filtration rate (eGFR)].

To estimate the association between sex and hyponatremia among diuretic users, it is necessary to include all types of diuretics and use serum sodium levels instead of hyponatremia coded as an adverse drug reaction. In addition, it is also important to adjust for relevant covariables, such as drug dose, comorbidities and comedication and several patient characteristics (Chow et al., 2003; Association NIVDI, 2012; KNMP, 2013; Rodenburg et al., 2013; Ramírez et al., 2019). The aim of this study is to find more evidence whether sex is associated with hyponatremia at hospital admission and whether there are risk differences for the different diuretic groups.

## Materials and methods

### Setting

A retrospective nested case-control study was carried out using data from the Tergooi MC, a general community teaching hospital with 500 beds, and Haga Teaching hospital, a top-clinical hospital with over 600 beds, in the Netherlands. Data for this nested case-control study were retrospectively extracted from the electronic health record (EHR) system between the 1st of January 2017 and the 31st of December 2021 for Tergooi Hospital and between the 1st of January 2017 and the 1st of July 2021 for Haga Teaching hospital. Patient selection and data collection were performed using a rule-based text-mining software (IQVIA Patient Finder Solution-CTcue B.V., Amsterdam, the Netherlands). Patient selection and data collection with this tool has been validated before (van Laar et al., 2020). The cohort consisted of all patients admitted for any reason, aged 18 years or older, with a sodium (mmol/L) measurement within 24 h of the admission and who used diuretics at admission. All patients who declined consent for the reuse of their data for research purposes were excluded.

### Case definition and controls

A patient was defined as a case when having hyponatremia based on a serum sodium level of <135 mmol/L within 24 h of admission. Patients with sodium levels ≥135 mmol/L were defined as controls.

### Drug exposure

Information about medication use was obtained from the medication file of the EHR system. In this file three types of prescriptions were available: verified as home medication, registered as home medication, and clinical medication. Medication verification is performed by the clinical pharmacy at admission to verify which medication the patient was using before and at admission. In some cases however home medication is registered by the doctor as being used at home without being verified by the clinical pharmacy. Clinical medication is prescribed during admission. The current use of diuretics was mainly obtained from the verified as home medication registration (59%). If the medication was not registered as verified, medication registered as home medication was used. Diuretic use was considered currently in use when it was registered as verified or home medication 30 days before to 2 days after the date of admission. In case of administrative duplicates of specific diuretics, the diuretics closest to the date of admission were selected.

Patients were defined as exposed to a particular group of diuretics using based on the Anatomical Therapeutical Chemical (ATC) codes: C03A for low-ceiling diuretics including thiazides, C03B for low-ceiling diuretics excluding thiazides, C03C for high-ceiling diuretics, C03D for aldosterone antagonists, C03E for a combination of diuretics and aldosterone antagonists and considered as two diuretic groups, and C03X for other diuretics (Whocc, 2021). The collected data on diuretic use consisted of dosage form, dose strength, dose unit and frequency.

### Covariables

The following covariables were assessed as potential confounders: age (years), BMI (kg/m2), systolic and diastolic blood pressure (mmHg), estimated glomerular filtration rate (calculated by using the Chronic Kidney Disease Epidemiology Collaboration (CKD-EPI) creatinine equation) (mL/min/1.73m2), serum potassium (mmol/L). Furthermore, the following comorbidities were considered as potential confounders; liver cirrhosis, diabetes mellitus (DM), Syndrome of inappropriate antidiuretic hormone secretion (SIADH), hypothyroidism, nephrotic syndrome, polydipsia, adrenal insufficiency. The comorbidities were considered current when it was registered within 1 year before the admission date. Also, the use of other hyponatremia inducing drugs was considered as a potential confounder. The following comedications were assessed as having an effect on serum sodium: selective serotonin reuptake inhibitors (ATC-code N06AB), venlafaxine (ATC-code N06AX16), duloxetine (ATC-code N06AX21), NSAIDs (ATC-code M01A), antipsychotics (ATC-code N05A), carbamazepine (ATC-code N03AF01), oxcarbazepine (ATC-code N03AF02), desmopressine (ATC-code H01BA02), valproic acid (ATC code: N03AG01, ACE inhibitors (ATC code: C09A, C09B), angiotensin II receptor blockers (ATC code: C09C, C09D), proton pump inhibitors (ATC code: A02BC), and antineoplastic and immunomodulating agents (ATC code: L) when it was registered as verified or home medication 30 days before to 2 days after the date of admission. Adjustment for dose was also performed per diuretic group (Association NIVDI, 2012; KNMP, 2013). The dose per drug group was defined by the prescribed daily dose divided by the defined daily dose (DDD: hydrochlorothiazide 25 mg, chlortalidone 25 mg, furosemide 40 mg, bumetanide 1 mg, spironolactone 75 mg, eplerenone 50 mg) (Methodology WCCfDS, 2023). If the clinical measurement for eGFR or serum potassium was not available on the day of admission, the closest clinical measurement was chosen, from 1 day before admission to 1 day after admission. If BMI was not available on the day of admission the closest value to admission was chosen with a maximum measurement of 1 year before admission.

### Statistical analysis

A logistic regression analysis was used to calculate odds ratios (OR) with 95% CIs as a proxy for the risk of hyponatremia for women versus men while using diuretics. The odds ratios were adjusted for the following potential confounding covariables; age (years), body mass index (kg/m2), potassium serum level (mmol/L), systolic blood pressure (mmHg), diastolic blood pressure (mmHg), estimated glomerular filtration rate (ml/min/1.73 m2), number of diuretics in use, number of other hyponatremia inducing drugs and comorbidities (yes/no). Stratified analyses were performed for the specific diuretic groups (thiazides, loop diuretics and aldosterone antagonists), and also adjusted for the potential confounding covariables including the dose for the diuretic group. Furthermore, stratified analyses were performed by severity of hyponatremia (severe: <125 mmol/L), mild 125–134 mmol/L) for all diuretics and stratified per diuretic group. Multiple imputation was used to adjust for missing measurements of covariables (BMI, serum potassium level, systolic blood pressure, diastolic blood pressure and eGFR). All statistical analyses were performed using IBM SPSS version 28.

## Results

In total, 2,506 patients were included in the present study, of whom 50.0% were women (n = 1,254). 20.6% of the diuretic using patients had hyponatremia upon admission. These cases were statistically significantly older than controls, had a lower BMI, lower systolic and diastolic blood pressure and a lower eGFR (Table 1). 56.2% of the cases were women. They were statistically significantly older, had a lower BMI, higher systolic blood pressure and lower potassium measurements than the male cases (Table 1).

**TABLE 1:** Baseline characteristics of cases and controls^a^.

	Cases^b^			Controls^c^		
	Total sample	Women	Men	Total sample	Women	Men
N (%)	516 (100.0)	290 (56.2)	226 (43.8)	1,990 (100.0)	964 (48.4)	1,026 (51.6)
Age (years), mean (SD)	77.9 (12.6)^d^	81.4 (11.0)^e^	73.4 (13.1)	76.6 (12.6)	79.4 (12.5)^f^	73.9 (12.0)
BMI (m2/kg), mean (SD)	27.1 (5.8)^g^	26.5 (6.3)^h^	27.9 (5.1)	27.8 (5.6)	27.6 (6.1)^i^	27.9 (5.2)
Systolic BP (mmHg), mean (SD)	141.9 (30.6)^j^	145.0 (28.6)^k^	137.9 (32.5)	145.7 (29.6)	147.7 (30.7)^l^	143.8 (28.4)
Diastolic BP (mmHg), mean (SD)	78.2 (17.6)^m^	77.6 (17.8)	79.0 (17.4)	81.4 (18.1)	80.6 (0.6)^n^	82.2 (17.6)
Sodium (mmol/L), mean (SD)	130.4 (4.4)	130.3 (4.5)	130.6 (4.2)	139.1 (2.7)	139.1 (2.7)	139.1 (2.6)
Potassium (mmol/L), mean (SD)	4.1 (0.7)	4.0 (0.7)^o^	4.2 (0.7)	4.0 (0.6)	4.0 (0.6)^p^	4.1 (0.6)
eGFR (ml/min/1.73 m2), mean (SD)	59.1 (26.1)^q^	59.1 (24.2)	59.2 (28.5)	61.7 (23.3)	60.7 (23.3)	62.6 (23.4)
Number of types of diuretics in use, mean (SD)	1.24 (0.5)	1.22 (0.5)	1.25 (0.5)	1.22 (0.4)	1.22 (0.4)	1.23 (0.4)
Number of comedications in use, mean (SD)	1.35 (0.9)	1.37 (0.9)	1.34 (0.9)	1.34 (0.9)	1.32 (1.0)	1.36 (0.9)
Comorbidities (N, %)	182 (35.3)^r^	91 (31.4)^s^	91 (40.3)	512 (25.7)	215 (22.3)^t^	297 (28.9)

^a^ See for number of patients with missing values Appendix 1.

^b^ Cases had a serum sodium level <135 mmol/L at hospital admission.

^c^ Controls had a sodium level ≥135 mmol/L at hospital admission.

^d^ Cases were statistically significantly older than controls (*p* = 0.011).

^e^ Female cases were statistically significantly older than male cases (*p* < 0.001).

^f^ Female controls were statistically significantly older than male controls (*p* < 0.001).

^g^ Cases had a statistically significantly lower BMI, than controls (*p* = 0.012).

^h^ Female cases had a statistically significantly lower BMI, than male cases (*p* = 0.001).

^i^ Female controls had a statistically significantly lower BMI, than male controls (*p* = 0.047).

^j^ Cases had a statistically significantly lower systolic blood pressure than controls (*p* = 0.006).

^k^ Female cases had a statistically significantly higher systolic blood pressure than male cases (*p* = 0.002).

^l^ Female controls had a statistically significantly higher systolic blood pressure than male controls (*p* = 0.012).

^m^ Cases had a statistically significantly lower diastolic blood pressure than controls (*p* < 0.001).

^n^ Female controls had a statistically significantly lower diastolic blood pressure than male controls (*p* = 0.045).

^o^ Female cases had a statistically significantly lower potassium measurement than male cases (*p* = 0.001).

^p^ Female controls had a statistically significantly lower potassium measurement than male controls (*p* < 0.001).

^q^ Cases had a statistically significantly lower eGFR, than controls (*p* = 0.045).

^r^ Cases had statistically significantly more often a comorbidity than controls (*p* < 0.001).

^s^ Female cases had statistically significantly less often a comorbidity than male cases (*p* = 0.036).

^t^ Female controls had statistically significantly less often a comorbidity than male controls (*p* = 0.001).

Women had a statistically significantly higher risk of hyponatremia than men while using diuretics in general (OR 1.37; 95% CI 1.12–1.66) (Table 2). The risk estimates increased when adjusted for age, BMI, potassium serum level, systolic blood pressure, diastolic blood pressure, eGFR, comorbidities, number of prescribed types of diuretics and number of other hyponatremia inducing drugs (ORadj. 1.55; 95% CI 1.22–1.98).

**TABLE 2:** ORs and adjusted ORs (ORadj) for women versus men.

	Cases		Controls		Unadjusted model			Adjusted model^a^		
	Women (n)	Men (n)	Women (n)	Men (n)	OR	95% CI		ORadj	95% CI	
Diuretics	290	226	964	1,026	1.37	1.12	1.66	1.55	1.22	1.98
Thiazide (like) diuretics: hydrochlorothiazide and chlortalidone	143	113	433	472	1.38	1.04	1.82	1.35	1.00	1.83
Loop diuretics: furosemide and bumetanide	145	94	503	481	1.48	1.11	1.97	1.62	1.19	2.19
Aldosterone antagonists: spironolactone and eplerenone	50	63	184	254	1.10	0.72	1.66	1.15	0.73	1.81

^a^ ORs, adjusted for age (years), BMI (kg/m2), potassium serum level (mmol/L), systolic blood pressure (mmHg), diastolic blood pressure (mmHg), eGFR (ml/min/1.73m2), comorbidities (yes/no), daily dose divided by the defined daily dose of the specific diuretic, number of prescribed types of diuretics and number of other hyponatremia inducing drugs (ATC, code: N06AB, N06AX16, N06AX21, N03AF, M01A, N03AG01,H01BA02, N05, C09A, C09B, C09C, C09D, A02BC, L) with the corresponding 95% CI.

Subanalyses were performed for the specific diuretic groups. The risk of hyponatremia at hospital admission was higher for women than for men for both use of thiazide diuretics or loop diuretics (OR 1.38; 95% CI 1.04–1.82 and OR 1.48; 95% CI 1.11–1.97 respectively). The risk among aldosterone antagonist users was also increased, but not statistical significant (OR 1.10; 95% CI 0.72–1.66). The higher risk for women while using thiazide diuretics or loop diuretics is also apparent after adjustment for the covariables including adjustment for dose of the specific diuretic (ORadj. 1.35; 95% CI 1.00–1.83 and ORadj. 1.62; 95% CI 1.19–2.19 respectively) (Table 2).

Subanalyses for the severity of the hyponatremia showed that the risk of mild hyponatremia at hospital admission was higher for women than for men (ORadj 1.36; 95% CI 1.10–1.68). The risk of severe hyponatremia was also statistically significantly higher for women versus men (ORadj 1.96; 95% CI 1.04–3.68) (Table 3).

**TABLE 3:** ORs and adjusted ORs (ORadj) for women versus men stratified for severity.

				Unadjusted model			Adjusted model^a^		
		Women (n)	Men (n)	OR	95% CI		ORadj	95% CI	
Diuretics	Severe hyponatremia^b^	29	17	1.82	0.99	3.33	1.96	1.04	3.68
	Mild hyponatremia^c^	261	209	1.33	1.09	1.63	1.36	1.10	1.68
	Normal serum sodium level^d^	964	1,026	ref.	ref.	ref.	ref.	ref.	ref.
Thiazide (like) diuretics: hydrochlorothiazide and chlortalidone	Severe hyponatremia	19	9	2.30	1.03	5.14	2.25	0.96	5.26
	Mild hyponatremia	124	104	1.30	0.97	1.74	1.27	0.93	1.74
	Normal serum sodium level	433	472	ref.	ref.	ref.	ref.	ref.	ref.
Loop diuretics: furosemide and bumetanide	Severe hyponatremia	13	6	2.07	0.78	5.50	2.28	0.81	6.45
	Mild hyponatremia	132	88	1.43	1.07	1.93	1.57	1.15	2.14
	Normal serum sodium level	503	481	ref.	ref.	ref.	ref.	ref.	ref.
Aldosterone antagonists: spironolactone and eplerenone	Severe hyponatremia	3	7	0.59	0.15	2.32	0.38	0.08	1.93
	Mild hyponatremia	47	56	1.16	0.75	1.78	1.22	0.76	1.95
	Normal serum sodium level	184	254	ref.	ref.	ref.	ref.	ref.	ref.

^a^ ORs, adjusted for age (years), BMI (kg/m2), potassium serum level (mmol/L), systolic blood pressure (mmHg), diastolic blood pressure (mmHg), eGFR (ml/min/1.73m2), comorbidities (yes/no), daily dose divided by the defined daily dose of the specific diuretic, number of prescribed types of diuretics and number of other hyponatremia inducing drugs (ATC, code: N06AB, N06AX16, N06AX21, N03AF, M01A, N03AG01,H01BA02, N05, C09A, C09B, C09C, C09D, A02BC, L) with the corresponding 95% CI.

^b^ Severe hyponatremia: serum sodium <125 mmol/L.

^c^ Mild hyponatremia: serum sodium 125–134 mmol/L.

^d^ Normal serum sodium level: serum sodium ≥135 mmol/L.

Within the thiazide diuretic users women had a statistically significantly higher risk of severe hyponatremia than men (OR 2.30; 95% CI 1.03–5.14) although after adjustment (ORadj. 2.25; 95% CI 0.96–5.26) not statistically significant anymore. An increased risk was also found for mild hyponatremia, although not statically significant (OR 1.30; 95% CI 0.97–1.74 and ORadj. 1.27; 95% CI 0.93–1.74).

Also for the loop diuretic users increased risks of severe hyponatremia were observed but not statistically significant (OR 2.07; 95% CI 0.78–5.50 and ORadj. 2.28; 95% CI 0.81–6.45). There was a statistically significantly higher risk of mild hyponatremia for women versus men (OR 1.43; 95% CI 1.07–1.93 and ORadj. 1.57; 95% CI 1.15–2.14).

The subanalyses for aldosterone antagonist users showed no statistically significant differences.

## Discussion

In this study, we found that women using diuretics have an almost 40%–60% increased higher risk of developing hyponatremia measured at hospital admission than men. After adjustment for age, BMI, potassium serum level, systolic blood pressure, diastolic blood pressure, eGFR, comorbidities, number of prescribed types of diuretics and number of other hyponatremia inducing drugs this risk increased to an almost 1.6 times higher risk.

This risk was observed for all the different diuretic groups, highest when using thiazide and loop diuretic users.

We were able to make a direct comparison between women and men and risk of hyponatremia at hospital admission. However, as described before the underlying mechanism for the higher risk of hyponatremia in women is still not fully understood (Hendriksen et al., 2023). There might be differences between women and men in the regulation of vasopressin and the activity and amount of several receptors and transporters. Sex differences in the arginine vasopressin (AVP) regulation were shown by hypotonic saline infusion that resulted in a higher renal excretion of sodium in women than in men while no differences were seen between phases of the menstrual cycle (Stachenfeld, 2014). It was suggested that testosterone has a greater influence on the renal sodium although this was not demonstrated. Also, in female rats the expression of the renal vasopressin receptors was higher and therefore could result in a higher sensitivity to AVP (Liu et al., 2011). The marketing authorization holder of desmopressin has advised to prescribe a 50% lower dose to women with nocturia based on the greater sensitivity of vasopressin in the renal tubules (Ferring, 2016). Based on a study with gonadectomized male rats there was no change in the antidiuretic effect of vasopressin whereas female rats had a decrease in urine flow and an increase in osmolarity (Grikinienė et al., 2004). Several differences in expression of receptors and transporters along the nephron were reported in mice, rats and humans (Grikinienė et al., 2004; Veiras et al., 2017; Hu et al., 2020). Women might have a greater number and activity of the Na/Cl co-transporters and a simulation model of the nephron suggested that activity of the Na/K/2Cl co-transporter is higher in women (Tahaei et al., 2020; Hu et al., 2021). All these differences, together with potential differences in water intake, could influence water balance and cause differences in risk of hyponatremia. In our previous study we showed a higher risk of hyponatremia related hospital admission (OR 1.86; 95% CI 1.64–2.11) in women than in men. However, in that study we did not have access to sodium serum levels. In this study we used sodium serum levels to define cases of hyponatremia instead of a diagnosis code that depends on the recognition and registration by the physician (Hendriksen et al., 2023). Furthermore, we were able to adjust for potential differences in BMI. Also, potassium serum levels, eGFR and systolic and diastolic blood pressure were available for the majority of the patients.

The previous study showed a higher risk than the current study (OR 1.37; 95% CI 1.12–1.66). This difference might be explained by the fact that hyponatremia is a known adverse drug reaction of diuretics and that physicians are increasingly more aware of the higher risk in women and therefore might be diagnosed more often with hyponatremia. Whereas in the current study we used serum sodium levels as an independent measure of hyponatremia. An electrolyte and kidney function blood panel upon admission is common practice and therefore the risk of difference in care between women and men is minimized. Using sodium measurements in included patients might explain the higher prevalence than in an outpatient setting (Andersson et al., 2024). Furthermore, the underlying conditions might be more severe in admitted patients and therefore influencing hyponatremia (Gankam-Kengne et al., 2013). The previous study might have comprised only the severe hyponatremia cases since the physicians had to diagnose hyponatremia and might not have registered mild hyponatremia serum sodium levels as a diagnosis. And in the current study we were not able to differentiate between an admission caused by hyponatremia or patients with hyponatremia. The adjusted OR for severe hyponatremia in the current study is more in accordance with the adjusted OR of the previous study (ORadj 1.96; ORadj 2.65 respectively). The subanalyses for severe hyponatremia were not statistically significant after adjustment but showed a trend towards a higher risk for both thiazide and loop diuretics. This might be due to a low number of patients within the severe hyponatremia subgroups.

In this study the use of loop diuretics showed a higher risk of hyponatremia in women than thiazide diuretic, whereas, in our previous study, the risk was higher when using thiazide diuretics (Hendriksen et al., 2023). Even though, based on the mechanism of action, loop diuretics do not tend to cause hyponatremia to the same extent as thiazide diuretics (Sterns, 2020). In both studies we were not able to differentiate only thiazide or only loop diuretic users due to small subgroups. This risk might be the result of the combination of diuretics although we adjusted for the number of diuretics used. Moreover, difference between the risk estimates may be confounded by the different indications, water intake or severity related to the specific diuretic. For example, heart failure is associated with hyponatremia and an indication for use of loop diuretics (Rodriguez et al., 2019). Therefore, we did not include heart failure as a comorbidity because that would have caused an overadjustment that could impact the accuracy of the estimates.

In the subanalyses with aldosterone antagonist users there was no statistically significant difference in risk between women and men. However, numbers were low. Other literature described no relevant associations between potassium and hyponatremia or it described an association with lower potassium values (Sharabi et al., 2002). When hyponatremia is chronic (onset over a period of >48 h), cells adapt to their hypo-osmolar environment by secreting potassium and intracellular osmolytes (KNMP, 2013). Furthermore, a large potassium deficit might contribute to hyponatremia with transcellular ion exchange (potassium exits and sodium enters the cells) (Chow et al., 2003). This could explain the influence of serum potassium on hyponatremia. Previous literature also described that thiazide-induced hyponatremia may be prevented by adequate potassium supplementation, for example, by combination with an aldosterone antagonist (Chow et al., 2003).

Our study has several strengths. Several exact measurements, coded diagnosis, and use of diuretics and other medication could be collected from the EHR-system of the hospitals. The use of the ranges for data collection on covariables gave us the opportunity to collect data around the day of admission. However, our study also has some limitations. Like many studies using real world data, our study had some missing measurements (serum potassium level, BMI, eGFR, systolic blood pressure and diastolic blood pressure). Consequently, multiple imputation was used to address this issue.

Furthermore, there was no information available on food intake, specifically sodium, and intake of water and alcohol. These parameters might affect sodium levels and could differ between women and men. Another limitation of this study was the inclusion of diuretic use registered by the doctor as home medication in the EHS when verified medication was not available. These diuretics might not be in use at the time of admission. This means that it is possible that some patients were incorrectly included as diuretic users in the study. Although, this is not expected to differ between women and men. In addition, the use of diuretics could be overestimated because of non-adherence. Not every patient uses their diuretics as prescribed by the doctor. For example, because they experience adverse drug reactions or forget to use their medication. Literature describes differences by sex in adherence of medication (Rolnick et al., 2013). However, a study on adherence of antihypertensive drugs showed no sex differences (Biffi et al., 2020).

In conclusion, sex is independently associated with hyponatremia among diuretic users also after adjustment for covariables. Women are more at risk for hyponatremia at hospital admission than men while using thiazide or loop diuretics. The underlying mechanism of this sex difference should be further investigated to be able to provide sex-specific recommendations of treatment in the future.

## Data Availability

The data analyzed in this study is subject to the following licenses/restrictions: The data that support the findings of this study are available from Tergooi MC and Haga Teaching Hospital but restrictions apply to the availability of these data, which were used under license for the current study, and so are not publicly available. Data are however available from the authors upon reasonable request and with permission of Tergooi MC and Haga Teaching Hospital. Requests to access these datasets should be directed to l.e.visser@hagaziekenhuis.nl.

## Appendix 1: Number of patients with missing values per covariable (%)

**Table:**

	Cases			Controls		
	Total sample	Women	Men	Total sample	Women	Men
BMI	150 (29.1)	81 (27.9)	69 (30.5)	410 (20.6)	232 (24.1)	178 (17.3)
Systolic BP	3 (0.6)	3 (1.0)	0 (0.0)	27 (1.4)	14 (1.5)	13 (1.3)
Diastolic BP	3 (0.6)	3 (1.0)	0 (0.0)	28 (1.4)	15 (1.6)	13 (1.3)
Potassium	16 (3.1)	8 (2.8)	8 (3.5)	32 (1.6)	14 (1.5)	18 (1.8)
eGFR	32 (6.2)	17 (5.9)	15 (6.6)	97 (4.9)	42 (4.4)	55 (5.4)

In total 105 female cases (36.2%), 87 male cases (38.5%), 289 female controls (30.0%) and 251 male controls (24.5%) had one or more missing values.
