# Supplementary material for: Women have a higher risk of hospital admission associated with hyponatremia than men while using diuretics
Source: Front Pharmacol. 2024 Aug 6;15:1409271. doi: 10.3389/fphar.2024.1409271 (PMC11333345; doi:10.3389/fphar.2024.1409271)
Supplement: Supplementary file 1 [file Table1.DOCX]

Appendix 1.

Number of patients with missing values per covariable (%)

|  | Cases | | | Controls | | |
| --- | --- | --- | --- | --- | --- | --- |
|  | Total sample | Women | Men | Total sample | Women | Men |
| BMI | 150 (29.1) | 81 (27.9) | 69 (30.5) | 410 (20.6) | 232 (24.1) | 178 (17.3) |
| Systolic BP | 3 (0.6) | 3 (1.0) | 0 (0.0) | 27 (1.4) | 14 (1.5) | 13 (1.3) |
| Diastolic BP | 3 (0.6) | 3 (1.0) | 0 (0.0) | 28 (1.4) | 15 (1.6) | 13 (1.3) |
| Potassium | 16 (3.1) | 8 (2.8) | 8 (3.5) | 32 (1.6) | 14 (1.5) | 18 (1.8) |
| eGFR | 32 (6.2) | 17 (5.9) | 15 (6.6) | 97 (4.9) | 42 (4.4) | 55 (5.4) |

In total 105 female cases (36.2%), 87 male cases (38.5%), 289 female controls (30.0%) and 251 male controls (24.5%) had one or more missing values.
